# The milk of human kindness: the story of the Mothers Milk Bank at Austin

**DOI:** 10.1186/1746-4358-1-6

**Published:** 2006-03-27

**Authors:** Barbara Wilson-Clay

**Affiliations:** 1 BS, IBCLC, Austin Lactation Associates,12710 Burson Drive, Manchaca, Texas, USA

## Abstract

Increased scientific study of human milk and awareness of the special nutritional needs of the premature infant have stimulated interest in human donor milk banking. Yet only three donor human milk banks existed in the United States in 1998. Having observed better outcomes in human milk-fed neonatal intensive care patients, two neonatologists in Austin, Texas, founded The Mothers Milk Bank at Austin (MMBA). Since opening in 1999, the MMBA has expanded rapidly as the result of careful planning, innovative procedures, fiscal stability, and widespread community support. The non-profit organizational structure, diversity and progressive vision of the board of directors and staff, and creative on-going public relations efforts have contributed to the success of the project. The MMBA demonstrates a model for 21^st ^century milk banking.

## Background

The nutritional superiority and immunological benefits provided by human milk are widely accepted [1,2]. Certain populations of high-risk infants may profit even more from these protections [3-7]. Ideally, own-mother's milk is provided. There is debate about whether banked human donor milk confers similar advantages. A systematic review examined outcomes of preterm infants fed donor human milk versus infant formula [8]. Meta-analysis indicated that the use of donor human milk was associated with a significant reduction in the relative risk of development of necrotizing enterocolitis (NEC) [8].

Recently, Schanler and colleagues questioned the benefit of banked human donor milk [9]. However, tables included in the Schanler study describe a significant decrease in chronic lung disease in the both own-mothers' milk and donor milk groups compared to the group receiving preterm infant formula [10]. Further, although the small sample size was inadequate to detect statistical significance, additional data included in the tables indicate that the rate of NEC was six per cent in the mothers milk and donor milk groups compared to 11 percent in the preterm infant formula group [10].

While un-pasteurized, own mothers' milk remains the gold standard, many mothers, particularly those who have given birth to preterm babies, have difficulty producing adequate volumes of milk [9,11-13]. In Austin, the capital of Texas, neonatologists George Sharpe, MD and Audelio Rivera, MD, observed in their neonatal intensive care units that human milk-fed infants experienced better outcomes and shorter hospitalizations. They became interested in establishing a human donor milk bank in order to provide human milk to infants whose own mothers were unable to fully nourish them.

Dr. Sharpe recalls, "I became enamored of the prospect of not having to treat NEC, one of the worst complications of the growing premature. A milk bank seemed an essential thing to do for our babies." Dr. Rivera agrees. "We started the milk bank to ensure that feedings for preterm infants would be safe and healthy, resulting in fewer complications." In 1998, doctors Rivera and Sharpe joined forces to found what would become the fourth US human donor milk bank, the first such facility to open since 1992. The ambitious scope of the project makes it a model for 21^st ^century milk banking.

**Figure 1:**
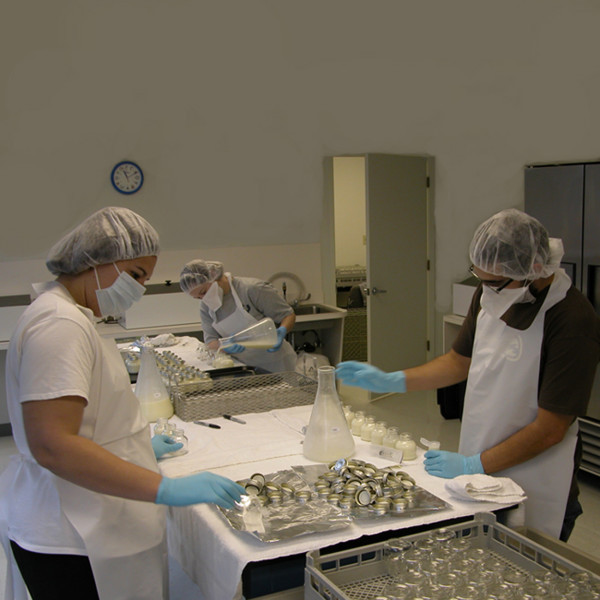
Pasteurization room at the Mothers Milk Bank at Austin.

**Figure 2:**
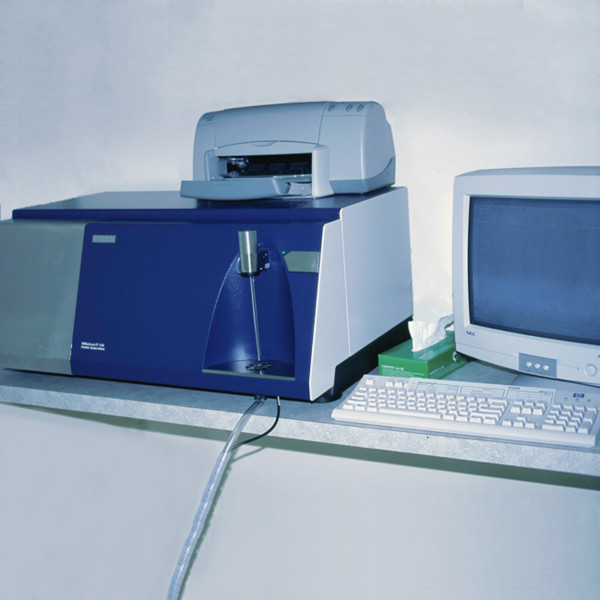
Foss Milkoscan.

## Discussion

### History of the project

Rivera and Sharpe held a series of exploratory meetings to investigate available resources and solicit community support. Their reputations were crucial to selling the idea of a new milk bank. Two local lactation consultants, a NICU nurse, several La Leche League leaders, a hospital chaplain, the mother of a preterm infant, and two additional neonatologists formed a core group. The group adopted an egalitarian working style, set family-friendly policies, pledged to support breastfeeding, and developed ethical guidelines for the project. Further, they agreed to embrace new technologies and to include a research component to the project in order to advance the science of human donor milk banking.

After touring existing milk banks and engaging in helpful dialog with the Human Milk Bank Association of North America (HMBANA), the group decided to organize the Austin milk bank as a community-based, free-standing facility. This crucial decision resulted from concerns that over-identification with any single institution would prevent acceptance from competing hospitals and limit widespread community support. Because historical review suggested that milk banks within existing institutions are vulnerable to funding cuts during budget crises, an autonomous milk bank with a supervising board of directors was deemed the most stable model for success. Additionally, the founders committed to the principle of non-profit milk banking in order to protect their ability to provide donor milk by prescription without regard for recipients' ability to pay. Once these organizational and philosophical decisions were made, the group constituted itself as a Board of Directors (BOD). In 1998, with pro bono assistance from a generous attorney, the BOD applied for and was granted status by the US Internal Revenue Service (IRS) as a 501(c)(3) nonprofit organization.

The Mothers Milk Bank at Austin (MMBA) owes much of its success to the camaraderie and diverse composition of the BOD, its exceptional staff, and to careful fiscal management. The project is funded by grants, donations, and, increasingly, by milk processing fees paid by hospitals that order supplies of milk for their preterm nurseries. The healthy financial status of the MMBA allows for expansion of services and continual up grading of equipment and methodologies.

The founding neonatologists currently serve as President and Vice President of the BOD. A vital liaison with the Austin Blood and Tissue Center was established when their president consented to serve as the MMBA's treasurer. The 21 member BOD meets eight times yearly to review operations, set policy, and continually reevaluate program goals. Initially, BOD members performed operational tasks. Staff now assumes total responsible for the day-to-day running of the milk bank. Consequently, such frequent board meetings are not essential. However, the creative input of the diverse fellowship comprised by the BOD is felt to be one of the cohesive elements guaranteeing the continuing success of the project.

Permanent board seats are designated for a representative from La Leche League, an IBCLC, and a public member (either a donor or a mother whose child had been a milk recipient). Another seat is designated for an ethics advisor. An Episcopal priest, a rabbi, and a Catholic nun have served in this capacity. The remaining board seats include representatives from the insurance, business, high tech, advertising, legal, arts and entertainment communities. The diversity of the BOD connects the milk bank project with funding and publicity resources that might otherwise not have been available, and provides a flexible mechanism for accessing valuable consulting expertise at no cost.

An early task of the MMBA was the creation of an informational website [14]. In 1999, a community open house helped familiarize local doctors, health care professionals, and the Austin community with the milk bank. A series of publicity events followed involving members of the local arts and music scene. Several outdoor concerts held annually on Mothers Day, and fund-raising events held in nightclubs and coffee houses raised awareness of the value of human milk and endeared the project to the community. While such events generally raised only small amounts of money (averaging US$5,000.) the publicity and raised community awareness led to increased private and corporate donations. Creative public relations strategies continue to attract donations of milk, money, and assist in recruiting volunteer workers.

In the spring of 1999, the BOD and the project's first Executive Director, Andrea Morgan, produced a Policies and Procedures manual, and adopted the milk banking standards and guidelines of HMBANA. The first milk donors were screened and approved following the HMBANA guidelines, (available on the HMBANA website) [15].

### Quality control

The MMBA adopted protocols for bacteriological screening, milk pasteurization, and nutritional labeling that met or exceeded HMBANA guidelines. Pre-pasteurization milk samples are drawn and plated for identification and counting of bacterial colonies. Milk testing positive for *Staphylococcus aureus*, methicillin- resistant *S. aureus*, and any of the *bacillus *species are discarded owing to the fact that endotoxins of these organisms are heat-resistant. Pasteurization may not render them harmless [16]. Aseptic technique is employed throughout the pasteurization process (see figure 1). Batches of three- and four-ounce (88–118 ml) bottles of milk are pasteurized using the long-term low temperature Holder technique, which holds milk at 62.5 °C for 30 minutes in a shaking water bath.

Milk samples are drawn and re-tested following pasteurization. No bacterial growth is tolerated in post-pasteurization milk. Pasteurized milk is held in freezers until results of post-pasteurization bacteriological testing are received. The milk is approved once pasteurized milk samples show no growth on 48-hour cultures plated and read by an independent microbiology laboratory. MMBA staff maintains careful records for each batch of processed milk, including statistics on what percentage of milk is discarded owing to contamination. All bottles and batches are labeled and numbered to permit tracking.

Pediatrix, a national organization of pediatricians and neonatologists, donated a Foss Milkoscan to the milk bank in 2000, allowing for nutritional analysis of pre- and post-pasteurization human donor milk (see figure 2). Widely used in the dairy industry, the Milkoscan uses a full spectrum infrared laser to conduct a nutritional analysis of the macronutrients found in milk – fat, protein, and lactose. Currently, no other non-profit US milk bank is utilizing Milkoscan technology. Calibrating the Milkoscan for human milk values took MMBA researchers many months [16]. Eventually, the MMBA was able to dispense milk labeled with specific calorie and protein values. During the calibration period, the Milkoscan sampling process revealed widely varying fat contents in the milk of donor mothers. While not an issue for their own, thriving babies, batches of low calorie milk were deemed inadequate for the growth needs of preterm infants. By 2003, the MMBA made the decision to move from random to target pooling to enable the milk bank to dispense labeled bottles of 20, 22, or 24-calorie milk.

By the end of 1999, the MMBA processed 10,000 ounces (296 litres) of milk and was dispensing milk to three hospitals and one infant in the community. By 2000, the staff grew to include a program director and a program assistant, who supervised all aspects of the donor milk program, including pasteurization. The addition of staff enabled increased enrollment of donors and increased pasteurization capacity. By the end of 2000, the MMBA had 148 donor mothers, and was serving six hospitals and nine outpatients clients from all over Texas. Over 51,000 ounces (1508 litres) of milk were pasteurized.

### Cost of MMBA donor milk

Human donor milk obtained from non-profit milk banks is not sold. A processing fee is charged to partially recover the expense of producing the milk. Production costs include donor screening and blood testing, microbiological testing of milk, pasteurization costs, storage costs, equipment and shipping expenses, staff salaries, and the expenses of operating the physical plant. Because actual production costs exceed the processing fees charged, human donor milk from the MMBA is partially subsidized by charitable donations and grants. The current processing fee charged for one ounce (28 ml) is US$3.25.

In January 2006, 14 non-hospitalized children were receiving milk from the MMBA. These recipients included infants who were discharged from the preterm nursery unable to tolerate infant formula. Others included older babies with nutritional handicaps. Approximately one half of these recipients are termed "charity patients". This means they pay a token fee or nothing for the milk. The MMBA absorbs these costs and raises donations from the community to defray them.

Approximately 90 per cent of MMBA milk recipients are hospitalized preterm infants. Generally, the cost of the milk provided to hospitals is recovered in the form of processing fees. Some milk is paid for by private insurance, and some families pay for all or part of their infant's supply. In Texas, some of the milk processing fees are paid for by Medicaid.

Medicaid is a US government-sponsored program that releases money to the states to help meet the medical needs of specialized groups of citizens, including low income families and children in foster care. A special provision of Medicaid allows preterm infants to be temporarily enrolled as recipients, providing help to families who would otherwise be overwhelmed by their medical costs. Rep. Glen Maxey, state representative from the MMBA's congressional district, successfully sponsored legislation in 2001 to obtain Medicaid funding to pay for human donor milk. The inclusion of Medicaid coverage for donor human milk made it possible to routinely secure donor milk for a wider variety of clients including hospitalized preterm infants without private insurance, outpatient babies with special nutritional needs, and infants in the Texas foster care system.

### Recent history and plans for future expansion

The BOD conducts annual strategic planning sessions to chart the course for each up-coming year. This encourages continual evaluation of the goals of the project. The BOD made an early decision to expand collection centers outside of the Austin area in order to keep pace with increasing demands for milk. A milk collection depot was opened in Houston in 2000, the first of nine collection sites around the state. Some hospitals in various US cities that were using MMBA donor milk began exploring the idea of starting their own milk banks. Since the founding of the MMBA, one milk bank has re-opened in Delaware, and four additional US human donor milk banks have been established in Ohio, Indiana, Iowa, and Ft. Worth, Texas, each with mentoring assistance from the MMBA.

In 2004, Dr. Rivera presented research on nutritional data collected at the MMBA to the American Association of Pediatrics conference in San Francisco. Several on-going research studies describing growth outcomes of infants receiving human donor milk await completion and publication.

Staff members of the MMBA have written a manual entitled *Starting a Donor Human Milk Bank: A Practical Guide *[17]. The guide includes a historical and research review of milk banking. It provides specific suggestions on creating budgets and by-laws, and addresses a variety of legal and organizational issues. The manual describes staffing and equipment requirements, funding resources, and provides a template for other communities wishing to access the operational model employed by the Austin project.

In January 2006, the MMBA consisted of a staff of eight, and a well-organized cadre of volunteers. In addition to the BOD, an advisory panel of medical doctors and researchers has been formed, providing an additional expert consulting body. Year-end figures for 2005 report that the MMBA dispensed 186,777 ounces (5524 litres) of milk, routinely supplying milk to 26 hospitals nationally.

The MMBA is outgrowing its current facility, which houses the staff, three pasteurizers, nine freezers (equipped with sensors and emergency back-up power sources), a commercial dishwasher, an ice-maker, and a small research laboratory. The BOD plans to launch a campaign to acquire funds for a new space. Ideally this will include a loading dock to better handle deliveries and shipments, expanded research facilities, and space for new, advanced pasteurization technologies.

## Conclusion

Inquiries and requests for mentoring assistance from Australia, Israel, Korea, and numerous American cities suggest a resurgence of interest in human donor milk banking. Based on a very successful first six years, the MMBA believes that the model of a non-profit, community-based milk bank is viable and sustainable. As new research and improved technologies provide better mechanisms for quality control, less loss of milk nutrients, and permit specific nutritional labeling, an expanded role in the use of human donor milk to provide a safety net for infants seems assured.

## Competing interests

Barbara Wilson-Clay is a co-founder and serves as an unpaid volunteer on the Board of Directors of The Mothers Milk Bank at Austin. Neither she, nor any member of the Board of Directors of the MMBA receives any income from any aspect of donor human milk banking.
